# Supplementary material for: Long-term changes in body image after bariatric surgery: An observational cohort study
Source: PLoS One. 2022 Dec 7;17(12):e0276167. doi: 10.1371/journal.pone.0276167 (PMC9728839; doi:10.1371/journal.pone.0276167)
Supplement: Attachment — Submitted filename: Response to reviewers.docx [file pone.0276167.s002.docx]

Manuscript PONE-D-21-30163

**Response to Reviewers**

Dear Editor and Reviewers,

Thank you for giving us the opportunity to submit a revised version of the manuscript “Long-term changes in body image after bariatric surgery : an observational cohort study” for publication in PLOS ONE.

We appreciate the time and effort that you and the reviewers dedicated to providing feedback on our manuscript and are grateful for the insightful comments on and valuable improvements to our paper. We have incorporated most of the suggestions made by the reviewers. Those changes are highlighted within the manuscript.

Please see below for a point-by-point response to the comments and concerns. All page numbers refer to the revised manuscript file with tracked changes.

Additional Editor Comments:

Dear Authors,
The article is interesting and valuable. However, there are some improvements to make:

1. Line 56: The authors mention that bariatric surgery also significantly improves most obese patients' comorbidities. Could you please detail (for example, diabetes, metabolic syndrome, hypertension..)?

We thank the reviewer for this pertinent comment. We detailed that in the manuscript.

2. It would be interesting if you could make some correlations between several factors and the change in body image over time, such as sex, age, the onset of obesity, type of BS, comorbidities, weight loss.

We thank the reviewer for this comment. We did not find any correlation between the factors you mentioned above and the change in body image over time.

3. Do you have separated the patients who achieved BMI <30? It could show different results between the groups. It is interesting to explain the results, for this cluster of patients .

As you requested, we had a look at patients according to their final BMI i.e > 30 or < 30 and we added a sentence in the manuscript (lines 171-174).

4. Please explain and extend the discussion about the reasons for the dropouts after 18 months.

As you suggested, we explained that in the discussion (lines 273-276).

5. Refresh the whole manuscript, correct miswriting, and check the punctuation.

We checked the whole manuscript to ensure that the text is optimally phrased and free from typographical and grammatical errors.

Reviewer #1: Dear Authors,

The aim of following patients' body images for a longer period after bariatric surgery is a very important point considering it may help to build strategies to achieve better and prolonged results, even if not using the most appropriate tools. Just wondering if you had separated the patients who achieved BMI <30 it could show different results between the groups. Also, I wondered if wouldn't be interesting to refer to the reasons for the dropouts after 18 months.

I suggest you review the writing as there are a few miswriting and check the punctuation.

Reviewer #2: The paper is an original article regarding the body image perception and its variation in patients that underwent bariatric surgery. The topic is extremely interesting. The paper is written in a fluid English language and based on a solid statistical analysis. Some issues could be improved before publication:

1. Line 56: The authors mention that bariatric surgery also bring important improvement in comorbidities that most obese patients have. Could you please detail (for example: diabetes, metabolic syndrom, hypertension..)?

We thank the reviewer for this pertinent comment. We detailed that in the manuscript.

2. It would be interesting if you could make some correlations between several factors and the change in body image over time, such as sex, age, onset of obesity, type of BS, comorbidities, weight loss

As explained before, we did not find any correlation between the factors you mentioned above and the change in body image over time.
