# Supplementary material for: Long-term changes in body image after bariatric surgery: An observational cohort study
Source: PLoS One. 2022 Dec 7;17(12):e0276167. doi: 10.1371/journal.pone.0276167 (PMC9728839; doi:10.1371/journal.pone.0276167)
Supplement: Attachment — Submitted filename: Response to reviewers 2.docx [file pone.0276167.s003.docx]

Dear Editor and Reviewers,

Thank you for giving us the opportunity to submit another revised version of the manuscript “Long-term changes in body image after bariatric surgery: an observational cohort study” for publication in PLOS ONE.

We appreciate the time and effort that you and the reviewers dedicated to providing feedback on our manuscript and are grateful for the insightful comments on and valuable improvements to our paper. We have incorporated most of the suggestions made by the reviewers. Those changes are highlighted within the manuscript.

Please see below for a point-by-point response to the comments and concerns. All page numbers refer to the revised manuscript file with tracked changes.

Reviewer #3: This manuscript reports on data that is almost a decade old, for reasons that are unclear. Since that time, research in this area has grown to a size larger than suggested here. Much of the referenced literature is dated and many more recent empirical papers and reviews on this topic were missed. Further, the Figure Rating Scale is no longer viewed as an appropriate measure of body image dissatisfaction. It certainly not validated for those with clinically severe obesity. The results of the MBSRQ-AS replicate those from other studies. So, it's not clear what this small, dated study adds to the literature.

Response:

Thank you for taking the time to review our article.

Indeed, our data are almost a decade ago since one of our goal was to follow patients during 5 years that means that the study started almost a decade ago.

We agree that the Figure Rating Scale has many limitations: limited range of figures are presented on the scale (it is indeed not a tool for subjects with super obesity), illustrations may not capture all the different body types, and validity may vary by ethnic group. Nevertheless, this tool was used frequently when we started collecting data and is still used today [58].

In addition, it has the advantage of being quickly administered, showing a strong correlation with the body mass index. It provides a simple, self-administered technique of collecting estimates of body dissatisfaction, and avoids variability that might be caused by language deficits.

An interesting option could have been the use of the Body-Image Assessment for obesity or BIA-O as a figural rating scale (the ﬁrst one adapted speciﬁcally for use in obesity) with the BODY-Q as already mentioned in the manuscript [59]. The BIA-O was however much less used than the FRS in the bariatric surgery literature.

We think that the current research could add to the literature because it is the only study with so many body image assessments 5 years after bariatric surgery. We added this information in the manuscript [53, 54].

We thank the reviewer for giving us the opportunity to read again the literature. We have now cited additional recent studies in our paper.

[28]	Paul MA, Opyrchał J, Knakiewicz M, Jaremków P, Duda-Barcik Ł, Ibrahim AMS, Lin SJ. The long-term effect of body contouring procedures on the quality of life in morbidly obese patients after bariatric surgery. PLoS One. 2020 Feb 21;15(2):e0229138.

[37]	Perdue TO, Schreier A, Swanson M, Neil J, Carels R. Majority of female bariatric patients retain an obese identity 18-30 months after surgery. Eat Weight Disord. 2020 Apr;25(2):357-364.

[53]	Mento C, Silvestri MC, Muscatello MRA, Rizzo A, Celebre L, Cedro C, Zoccali RA, Navarra G, Bruno A. The role of body image in obese identity changes post bariatric surgery. Eat Weight Disord. 2022 May;27(4):1269-1278

[54]	Bertoletti J, Galvis Aparicio MJ, Bordignon S, and Marceli Trentini C. Body Image and Bariatric Surgery: A Systematic Review of Literature.Bariatric Surgical Practice and Patient Care. Jun 2019.81-92.

[58]	Parzer V, Sjöholm K, Brix JM, Svensson PA, Ludvik B, Taube M. Development of a BMI-Assigned Stunkard Scale for the Evaluation of Body Image Perception Based on Data of the SOS Reference Study. Obes Facts. 2021; 14(4):397-404.

[59]	Williamson DA, Womble LG, Zucker NL, Reas DL, White MA, Blouin DC, Greenway F. Body image assessment for obesity (BIA-O): development of a new procedure. Int J Obes Relat Metab Disord. 2000 Oct;24(10):1326-32.

Reviewer #4:

I find the study interesting. The manuscript is well organized, and statistically well prepared. Maybe the idea is not new, and the outcome is similar to the other studies, even the one presented by my team in 2020 the long-term effect of body contouring procedures on the quality of life in morbidly obese patients after bariatric surgery

doi.org/10.1371/journal.pone.0229138.

The strength of the study is long followed for up to 5 years. The response rate of the study is 66% which is more than needed to assess as a valid study. One of the most important parts of the manuscript is to show that after 5 years there is a decline in the body assessment, but it is still higher than before bariatric surgery. So we can say that the patient qualification process was proper.

There is my summary

1. The authors have responded properly to the reviewers

2. The study is valuable due to the long follow up

3. The response rate is 66% - enough to publish

4. Would be better to have a multicenter study, but thanks to long follow-up it should be considered valuable.

5. Please add the mentioned study

6. In the next studies I would recommend the assessment of the body reception and psychiatric evaluation of the patients.

Response :

Thanks for your comment. Your study using the BODY-Q is very interesting and we have added your reference to our manuscript at line 211 [28].
